# Comparative analysis of KRAS codon 12, 13, 18, 61, and 117 mutations using human MCF10A isogenic cell lines

**DOI:** 10.1038/srep08535

**Published:** 2015-02-23

**Authors:** Britta Stolze, Stefanie Reinhart, Lars Bulllinger, Stefan Fröhling, Claudia Scholl

**Affiliations:** 1 Department of Internal Medicine III, Ulm University, Ulm, Germany; 2 Department of Translational Oncology, National Center for Tumor Diseases (NCT), German Cancer Research Center (DKFZ), Heidelberg, Germany; 3 Heidelberg University Hospital, Heidelberg, Germany

## Abstract

KRAS mutations occur in one third of human cancers and cluster in several hotspots, with codons 12 and 13 being most commonly affected. It has been suggested that the position and type of amino acid exchange influence the transforming capacity of mutant KRAS proteins. We used MCF10A human mammary epithelial cells to establish isogenic cell lines that express different cancer-associated KRAS mutations (G12C, G12D, G12V, G13C, G13D, A18D, Q61H, K117N) at physiological or elevated levels, and investigated the biochemical and functional consequences of the different variants. The overall effects of low-expressing mutants were moderate compared to overexpressed variants, but allowed delineation of biological functions that were related to specific alleles rather than KRAS expression level. None of the mutations induced morphological changes, migratory abilities, or increased phosphorylation of ERK, PDK1, and AKT. KRAS-G12D, G12V, G13D, and K117N mediated EGF-independent proliferation, whereas anchorage-independent growth was primarily induced by K117N and Q61H. Both codon 13 mutations were associated with increased EGFR expression. Finally, global gene expression analysis of MCF10A-G13D versus MCF10A-G12D revealed distinct transcriptional changes. Together, we describe a useful resource for investigating the function of multiple KRAS mutations and provide insights into the differential effects of these variants in MCF10A cells.

Under physiological conditions, the small GTPase KRAS is activated in a controlled manner through the exchange of GDP for GTP upon binding of growth factors to their receptors, which then allows KRAS to bind and activate various effectors mediating a multitude of cellular effects. For example, the well-studied PI3K-PDK1-AKT, RAF-MEK-ERK, and TIAM1-RAC1 cascades regulate cell survival, cell proliferation, and cytoskeletal organization, respectively^1^.

Point mutations in the *KRAS* gene occur in approximately 30% of human cancers and are particularly common in adenocarcinomas of the pancreas, lung, and colon^2^. Thus far, mutant KRAS is considered an undruggable target^3^,^4^,^5^, although new approaches for blocking KRAS activity continue to be developed^6^,^7^, and serves as a predictor of non-responsiveness to molecularly targeted therapies such as EGFR inhibitors in lung and colon cancer^8^,^9^.

On the molecular level, KRAS mutations result in reduced intrinsic GTPase activity, which in turn leads to permanent activation of KRAS itself and downstream signalling pathways, thereby mediating malignant transformation. These single amino acid substitutions typically affect hotspots at codons 12 and 13. However, KRAS mutations also occur in codons 18, 61, 117, and 146 at low frequencies^10^. Of note, there is evidence that the type of KRAS mutation determines their biochemical activity and transforming capacity. For example, experiments with murine NIH/3T3 cells showed that codon 12 mutations protected from apoptosis and promoted anchorage-independent growth more strongly than codon 13 mutations^11^, and KRAS-K117N and A146T are associated with lower levels of GTP-bound RAS compared to G12D in transfected HEK293FT cells^12^. Of potential clinical relevance, patients with colorectal cancer harbouring G13D mutations were reported to respond better to anti-EGFR therapy compared to patients with G12D mutations^13^,^14^, but the biological basis for this observation is currently unclear.

We hypothesized that a systematic comparative analysis would greatly improve our understanding of the differential effects of diverse KRAS mutations on signalling pathways, cellular functions, and possibly clinical outcomes. Thus far, most *in vitro* studies have been performed in murine or human cell lines overexpressing the RAS mutants under investigation, which makes it difficult to assign specific biochemical or cellular effects to the respective mutation itself, since overexpression of wildtype (WT) KRAS also has transforming properties^15^. In addition, it remains elusive whether the specific amino acid that replaces glycine at positions 12 and 13 has an influence on the biological effects of mutant KRAS.

To address these questions, we generated isogenic MCF10A human mammary epithelial cell lines harbouring WT KRAS and eight different KRAS mutations that were all expressed at close-to-endogenous levels, and analysed these cell lines for KRAS activity, activation of downstream signalling pathways, and various cellular phenotypes including morphology, proliferation, anchorage-independent growth, and migratory properties. The different mutations clearly varied in their ability to mediate biochemical and cellular responses, and overall caused only moderate effects compared to oncogenic *KRAS* alleles expressed at supraphysiologic levels.

## Results and Discussion

### Establishment of KRAS mutant MCF10A isogenic cell lines

**Figure 1:**
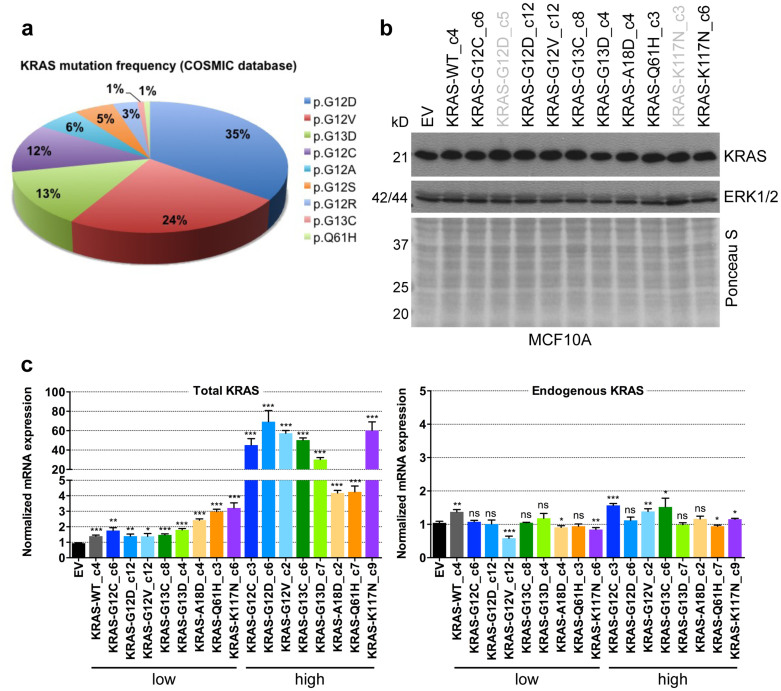
Generation of MCF10A isogenic cell lines harbouring various KRAS mutations. (a) Frequency of KRAS mutations across human cancers according to the Catalogue of Somatic Mutations in Cancer (COSMIC; http://cancer.sanger.ac.uk/cancergenome/projects/cosmic). Not depicted are KRAS-A18D and K117N, which occur in less than 1%. (b) Western blot analysis of KRAS protein expression in MCF10A cells transduced with EV and MCF10A clones expressing WT KRAS or the indicated KRAS mutations. Clones marked in black were used for further experiments. ERK1/2 expression and Ponceau S staining indicate equal loading. (c) Total (endogenous and exogenous, left panel) and endogenous (right panel) *KRAS* mRNA expression of MCF10A clones expressing low and supraphysiological (high) levels of the introduced KRAS mutations (triplicate; mean ± SD). P values were calculated relative to EV using an unpaired t-test. *, P < 0.05; **, P < 0.01; ***, P < 0.0001; ns, not significant.

To investigate the biochemical and functional consequences of different KRAS mutations, we chose the immortalised human mammary epithelial cell line MCF10A, since these cells are well characterised and represent a useful tool for assessing the transforming activity of oncogenes, such as *RAS*, *ERBB2*, and *PLK4*^16^,^17^,^18^. We introduced the four most frequent KRAS mutations (G12D, G12V, G13D, G12C), which account for 83% of all KRAS mutations, KRAS-G13C as the second most common alteration of codon 13, three rare mutations (Q61H, A18D, K117N), and WT KRAS into MCF10A cells by lentiviral transduction (Figure 1a).

Six to eleven clones per cell line were obtained by seeding single cells in 96-well plates, and KRAS protein levels were compared to those of MCF10A cells transduced with an empty control vector (EV) (Figure S1). Clones with KRAS protein levels comparable to those of EV-transduced cells were selected, and side-by-side analysis by western blotting confirmed similar expression levels in all KRAS mutant clones and EV-transduced control cells (Figure 1b). In all clones, the presence of the respective KRAS mutation was confirmed by Sanger sequencing (data not shown).

To quantify the expression of the introduced cDNAs, we measured total (endogenous and exogenous) and endogenous *KRAS* mRNA levels by quantitative RT-PCR. In the KRAS mutant clones, we observed slightly increased levels of total *KRAS* mRNA with 1.5 to 3-fold higher expression compared to EV-transduced control cells (Figure 1c, left panel). For comparison, we analysed clones with substantially higher KRAS protein levels (Figure S1), resembling the high expression typically seen in, e.g., NIH/3T3 or HEK293 cells transduced or transfected with *KRAS* cDNAs that are often used in functional studies. *KRAS* mRNA expression in these cells was increased up to 60-fold compared to control cells, illustrating that forced expression of mutant cDNAs using viral vectors often results in supraphysiologic KRAS levels, which complicates assessment of the biochemical and functional consequences. Endogenous *KRAS* mRNA levels remained unchanged in both low and high-expressing clones, indicating that MCF10A cells did not downregulate endogenous KRAS as compensatory mechanism (Figure 1c, right panel).

### GTPase activity of KRAS mutants

**Figure 2:**
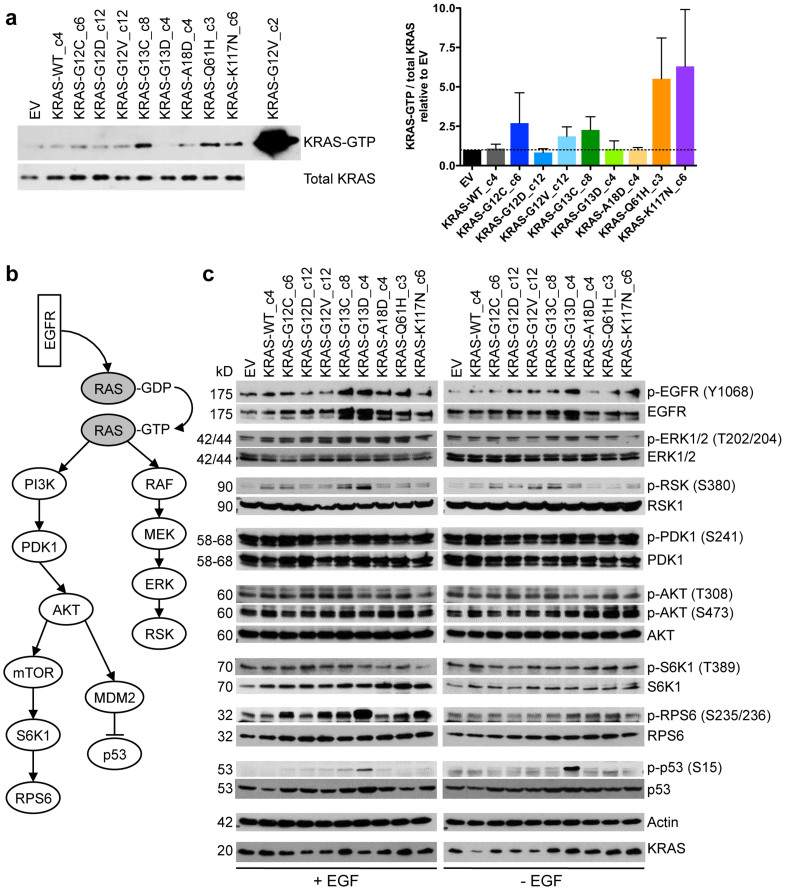
GTPase activity and signal transduction properties of KRAS mutants. (a) Immunoprecipitation of GTP-bound KRAS from EV-transduced MCF10A cells or MCF10A clones expressing the indicated KRAS mutants at low levels following stimulation with EGF (20 ng/ml) for 10 minutes after overnight starvation. A clone overexpressing MCF10A-G12V served as positive control. Shown are one representative blot (left panel) and the normalized KRAS-GTP levels from three independent experiments (right panel; mean ± SEM). (b) Scheme of the signalling pathways studied. (c) Western blot analysis of MCF10A clones expressing low levels of different KRAS mutations and cultured in regular growth medium (+EGF) or without EGF overnight (−EGF). Actin expression indicates equal loading.

To characterise the different KRAS mutants, we first measured their activity by determining KRAS-GTP levels in pulldown experiments with the RAS-binding domain of RAF1 as bait in MCF10A cells starved overnight and either left untreated or stimulated with EGF for 10 minutes. Under starved conditions, we detected no GTP-bound KRAS in MCF10A cells expressing the different KRAS variants at low levels (data not shown). Following EGF stimulation, we observed minor, non-significant effects of the different KRAS variants on activated KRAS in low-expressing clones (Figure 2a). MCF10A cells expressing WT KRAS or the mutants G12D, G13D, and A18D showed similar KRAS-GTP levels as EV-transduced cells, pointing to unchanged GTPase activity. In contrast, the exchange of glycine (G) for cysteine (C) at codons 12 and 13 and valine (V) at codon 12 increased the level of GTP-bound KRAS up to 2-fold, indicating that the identity of the replacing amino acid determines the GTPase activity of oncogenic KRAS, with aspartic acid (D) having the least effect. Unexpectedly, cells expressing the rare mutations Q61H and K117N showed the highest levels of KRAS-GTP with a 5 to 6-fold increase compared to EV-transduced cells. These results reveal variable GTP binding properties among KRAS mutants, which may confer different biological functions.

### Signal transduction properties of KRAS mutants

To analyse the ability of KRAS mutants to activate downstream signalling pathways, we examined the phosphorylation of EGFR, ERK, RSK, PDK1, AKT, p53, and components of the mTOR-S6K1-RPS6 axis in starved and unstarved MCF10A clones (Figure 2b). The effects of different variants expressed at near-physiologic levels were overall surprisingly modest. Compared to MCF10A-EV and MCF10A-WT cells, none of the cell lines expressing mutant KRAS at low levels showed increased phosphorylation of ERK, PDK1, and AKT under starved or unstarved conditions (Figure 2c). As expected, phosphorylated ERK and AKT were readily detectable in MCF10A clones expressing mutant KRAS at high levels (Figure S2). Activation of the mTOR signalling cascade, as determined by phosphorylation of S6K1 and RPS6, was not observed under starvation, whereas addition of EGF to the low-expressing clones or cells overexpressing mutant KRAS resulted in pronounced phosphorylation of RPS6, most prominently in the context of KRAS-G13D and K117N (Figure 2c and S2). However, given the lack of PDK1 and AKT phosphorylation, activation of RPS6 was likely not induced by the PI3K-AKT-mTOR-S6K1 cascade. Instead, MCF10A-G13D cells showed increased RSK1 phosphorylation upon EGF stimulation, suggesting RSK1 as the kinase that phosphorylates RPS6 in this genetic context. In support of this hypothesis, it has been shown previously that RSK can phosphorylate RPS6 at S235 and S236 in response to serum, growth factors, and oncogenic RAS in an mTOR-independent manner^19^. Since we did not observe activated RSK1 in MCF10A-K117N cells, RPS6 phosphorylation is likely mediated by other signalling proteins in these cells.

Interestingly, we observed increased expression of total and phosphorylated EGFR in response to both codon 13 mutations in MCF10A cells cultured in regular growth medium, an effect that was also evident under starved conditions in MCF10A-G13D cells (Figure 2c). Furthermore, MCF10A-G13D cells displayed strong phosphorylation of p53 at serine 15, a site that is known to be phosphorylated by ATM, suggesting induction of DNA damage response signalling by G13D-mediated replicative stress^20^. The effects of KRAS-G13D on EGFR and p53 were strikingly different compared to mutations of codons 12, 18, 61, and 117, and could provide a biological explanation for the favourable clinical outcome of patients with G13D-positive colorectal cancer receiving anti-EGFR therapy compared to patients with codon 12 mutations^13^,^14^. For example, it is conceivable that higher EGFR levels provide a larger contact surface for therapeutic antibodies targeting EGFR, such as cetuximab and panitumumab, and that the activated senescence program predisposes EGFR^high^ cells to death triggered by EGFR blockade. In accordance with this hypothesis, we observed increased sensitivity of MCF10A-G13D cells towards the EGFR inhibitors erlotinib and gefitinib compared to MCF10A-EV, G12D and K117N cells with 2 to 3-fold reduced IC50 values (Figure S3). To investigate this hypothesis further, correlating pretherapeutic EGFR and phospho-p53 levels with outcome in patients undergoing anti-EGFR therapy will be of interest.

### Effects of KRAS mutants on cell morphology

**Figure 3:**
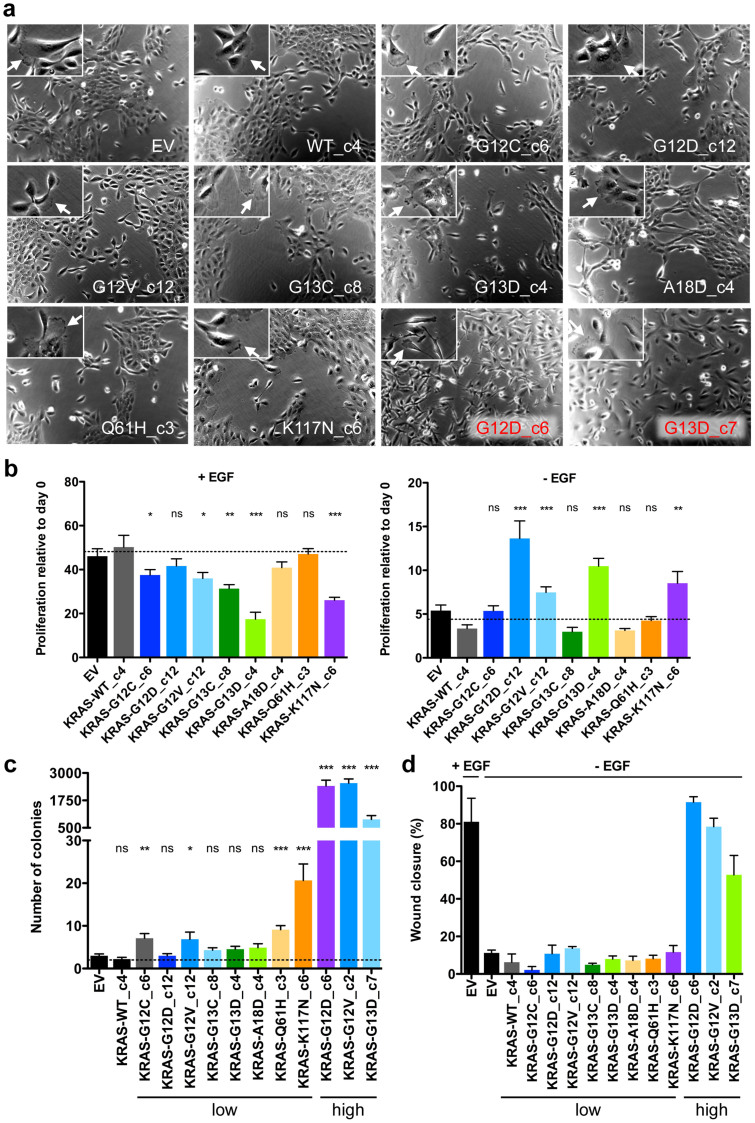
Cellular effects of KRAS mutants. (a) MCF10A clones expressing the indicated KRAS mutations at low (white letters) and high (red letters) levels were grown in regular medium to 50% confluency. Representative phase contrast micrographs (original magnification, 10×) are shown. Insets show 2-fold magnified details of the corresponding photographs, highlighting cells with lamellopodia formation (arrows). (b) Cell viability and proliferation of MCF10A clones expressing different KRAS mutations and cultured with and without EGF for four days, relative to the mean viability and proliferation of EV-transduced cells and cells expressing WT KRAS (dotted line). Results of three independent experiments performed in triplicate are shown (mean ± SEM). P values were calculated using an unpaired t-test. (c) Anchorage-independent growth in soft agar of MCF10A cells expressing different KRAS mutations at low and high levels, relative to colony formation of EV-transduced cells. Shown are colony numbers after six weeks (two to three independent experiments performed in triplicate, mean ± SEM). P values were calculated using an unpaired t-test. (d) Migration and wound healing of MCF10A cells expressing different KRAS mutations at low and high levels. Shown is the percentage of wound closure 24 hours after scratching a confluent monolayer (three independent experiments, mean ± SEM). *, P < 0.05; **, P < 0.01; ***, P < 0.0001; ns, not significant.

We next examined whether the introduction of mutant KRAS into MCF10A cells induces changes in cell morphology. Subconfluent MCF10A cells grow as clusters, and cells display cell protrusions and lamellipodia at the edges under normal growth conditions^16^. This phenotype was not altered by any of the eight different KRAS mutations expressed at near-physiological levels (Figure 3a). In contrast, high levels of KRAS-G12D and G13D resulted in marked morphological changes, as the formation of cell clusters was disrupted and the cells showed a fibroblastic and spindle-like shape lacking epithelial polarisation (Figure 3a), a phenotype that is characteristic for epithelial-mesenchymal transition^21^.

We also investigated the influence of EGF depletion on cell morphology according to KRAS mutation status. As in MCF10A-EV and MCF10A-WT cells, EGF withdrawal in MCF10A cells expressing low levels of mutant KRAS led to rounded cell clusters that completely lacked lamellipodia, and the cells assumed a cobblestone-like morphology similar to the changes observed under confluent growth conditions (Figure S4). In contrast, EGF depletion had less or no effect on the morphology of MCF10A cells overexpressing mutant KRAS, which appeared as single elongated cells with unaffected protrusions. These observations indicate that none of the KRAS mutations studied is able to induce morphological changes in MCF10A cells when expressed at physiological levels.

### Effects of KRAS mutants on survival and proliferation

It is known that the withdrawal of medium additives is detrimental for the survival and proliferation of MCF10A cells^22^. Our experiments indicated that overexpression of mutant KRAS is able to rescue MCF10A cells from EGF depletion, whereas cells expressing KRAS mutations at physiological levels remain reliant on EGF supplementation. To further test this, we analysed the ability of different KRAS mutants to confer EGF-independent growth to MCF10A cells. Expression of the three most common KRAS mutations (G12D, G12V, G13D) or the rare K117N allele was associated with significantly increased proliferation compared to EV or WT controls, whereas the other variants were not able to compensate for EGF withdrawal (Figure 3b, right panel). When cultured in the presence of EGF, cells expressing G13C, G13D, K117N, and, to a lesser extent, codon 12 mutations, showed reduced proliferation. This finding might suggest that constitutive KRAS activity in combination with EGF leads to excessive stimulation of signalling pathways and enhanced oncogene-induced senescence, particularly in the context of KRAS-G13D and K117N. In accordance with this assumption, we found increased senescence, as measured by β-galactosidase staining, in MCF10A-G13D and K117N cells (and to a lesser extent MCF10A-Q61H, G12C, G12D, and G13C cells) compared to MCF10A-EV and WT cells grown in EGF-containing medium (Figure S5).

### Effects of KRAS mutants on anchorage-independent growth

Next, we were interested in the impact of different KRAS mutations on anchorage-independent growth, an important characteristic of transformed epithelial cells. MCF10A cells exhibit several features of normal breast epithelium, including lack of colony formation in soft agar as a measure of anchorage-independent growth^22^. Compared to MCF10A clones overexpressing KRAS-G12D, G12V, and G13D, which gave very high colony numbers in soft agar, physiological expression of the different KRAS variants had only a weak effect on colony formation (Figure 3c). Whereas KRAS-G12D, G13C, G13D, and A18D did not confer anchorage-independent growth, slightly increased colony numbers were observed, in descending order, with cells expressing KRAS-K117N, Q61H, G12V, and G12C. This correlates with our observation that these clones exhibit the highest levels of GTP-bound KRAS (Figure 2a).

### Effects of KRAS mutants on migration

Since enhanced cell migration is required for tumour cell invasion and metastasis, we examined the motility of EGF-deprived MCF10A clones in a monolayer wound healing assay. In contrast to MCF10A cells overexpressing KRAS-G12D, G12V, or G13D, which were able to close the wound almost entirely after 24 hours, none of the KRAS mutations expressed at physiological levels conferred increased migratory abilities compared to EV or WT controls (Figure 3d and S6). Similar to cells overexpressing mutant KRAS, EV-transduced control cells reduced the wound area by nearly 80% when EGF was added (Figure 3d and S6).

### Transcriptional changes induced by KRAS mutants

**Figure 4:**
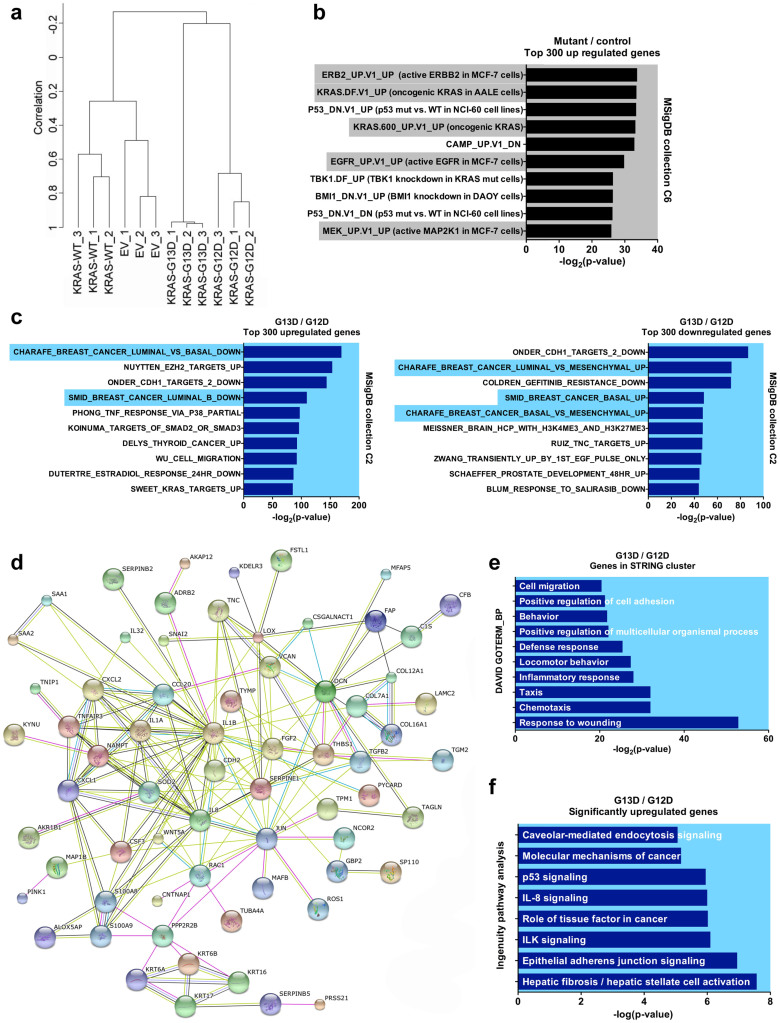
Transcriptional changes induced by KRAS mutants. (a) Unsupervised hierarchical cluster analysis (average linkage) based on 2,487 differentially expressed genes. Samples 1–3 indicate biological replicates. (b) The top 300 upregulated genes in KRAS mutant (G12D and G13D) versus control (WT KRAS and EV) samples were used for GSEA against the C6 oncogenic signatures collection of the MSigDB. Shown are the ten most significantly enriched gene signatures. Signatures associated with activated RAS signalling are highlighted in grey. (c) The top 300 upregulated (left panel) and downregulated (right panel) genes in MCF10A-G13D_c4 versus MCF10A-G12D_c12 samples were used for GSEA against the C2 curated gene sets of the MSigDB. Signatures associated with the luminal and basal/mesenchymal subtypes of breast cancer are highlighted in blue. (d) The top 300 upregulated genes in MCF10A-G13D_c4 versus MCF10A-G12D_c12 samples were analysed using the STRING software tool. Shown is the main cluster connecting 87 genes. (e) Query of the 87 genes indentified in d against the Gene Ontology Term (GOTERM) “Biological Process (BP)” using the DAVID bioinformatics tool. Shown are the ten most significant processes. (f) Ingenuity pathway analysis with all genes significantly upregulated in MCF10A-G13D_c4 versus MCF10A-G12D_c12 samples. Shown are the eight most significantly enriched pathways.

To better understand the differential effects of KRAS-G12D versus G13D on signalling pathways, which might provide insights into the unequal clinical response to anti-EGFR therapy associated with these genotypes^13^,^14^, we used Illumina HumanHT-12 Expression BeadChips to profile gene expression in MCF10A-EV cells and MCF10A clones expressing physiological levels of WT KRAS, KRAS-G12D, and KRAS-G13D. Unsupervised hierarchical cluster analysis based on 2,487 genes demonstrated that WT KRAS-expressing cells were more similar to MCF10A-EV cells than cells expressing KRAS mutants, which clustered separately (Figure 4a). Notably, KRAS-G12D and KRAS-G13D-expressing cells exhibited distinct gene expression profiles that clearly separated them not only from EV-transduced cells and cells expressing WT KRAS, but also from each other.

Class comparison analysis of control (KRAS-WT, KRAS-EV) versus KRAS mutant (KRAS-G12D, KRAS-G13D) samples demonstrated that both mutations caused widespread changes in the transcriptome of MCF10A cells. Using a 1.3-fold cutoff and a P value of 0.05, we found 939 genes to be significantly upregulated and 650 genes to be significantly downregulated (Table S1). To explore whether the transcriptional changes induced by KRAS-G12D and G13D in MCF10A cells were related to activated RAS signalling, we performed gene set enrichment analysis (GSEA) with the top 300 upregulated genes using the C6 oncogenic signatures collection of the Broad Institute Molecular Signatures Database (MSigDB; http://www.broadinstitute.org/gsea/msigdb/index.jsp). We identified several gene signatures derived from cells in which the RAS signalling pathway was activated through different stimuli, such as activation of ERBB2, EGFR, MEK, or KRAS itself (Figure 4b), again confirming the validity of our isogenic cell line models.

We next wanted to identify genes and pathways that are differentially regulated by KRAS-G12D and G13D. Class comparison analysis identified 1,207 significantly upregulated and 1,011 significantly downregulated genes in MCF10A-G13D cells versus MCF10A-G12D cells (Table S2). GSEA of the top 300 upregulated and the top 300 downregulated genes using the C2 curated gene sets of the MSigDB identified signatures from two independent studies that determined the gene expression profiles of luminal and basal/mesenchymal breast cancer^23^,^24^, in which KRAS-G13D was associated with the basal/mesenchymal subtype and KRAS-G12D with the luminal subtype (Figure 4c). Genes that were described to be upregulated in the basal subtype^23^ were highly expressed in MCF10A-G13D compared to MCF10A-G12D, including several cytokeratins (*KRT6A*, *KRT6B*, *KRT16*, *KRT17*), integrins (*ITGA6*, *ITGB4*), and others such as *LAMB3*, *LAMC2*, *ANXA8*, and *COL17A1* (Table S2). Similarly, MCF10A-G13D cells showed high expression of genes upregulated in the closely related mesenchymal subtype^23^, including collagens (*COL8A1*), proteases (*CTSC*, *PLAU*, *PLAUR*, *SERPINE1*, *SERPINE2*), and others such as *VIM* and *FN1* (Table S2). Although the basal subtype of breast cancer is associated with unfavourable clinical outcome^25^, it has been reported that breast basal-like cell lines are more sensitive to anti-EGFR treatment than luminal cell lines *in vitro*^26^. This differential sensitivity is reminiscent of the clinical behaviour of colorectal cancers harbouring KRAS-G13D versus G12D^13^,^14^ and suggests that KRAS-G13D induces distinct transcriptional and consequently biological changes that sensitise cancer cells of various tissue origin to anti-EGFR therapy.

In a second approach, we queried the STRING database (http://string-db.org) using the top 300 genes upregulated in MCF10A-G13D versus MCF10A-G12D cells to identify mutation-specific signalling networks. Of the 300 genes, 87 were clearly connected in one large cluster, suggesting activation of a distinct biological process (Figure 4d and Table S2). A search of the 87 genes against the Gene Ontology term “Biological Process” using the DAVID bioinformatics tool (http://david.abcc.ncifcrf.gov/home.jsp) revealed several processes that are associated with cytokine-induced cell migration (Figure 4e).

The top upregulated cytokine genes in MCF10A-G13D cells were *CXCL1*, *IL1B*, and *IL8* with a more than 10-fold increase in transcription compared to MCF10A-G12D cells (Table S2). Cytokines have been implicated in RAS-driven cancer previously, as activated RAS positively regulates the expression of cytokines to induce autocrine and paracrine signals that promote tumourigenesis^27^. For example, CXCL1 (also known as Gro-1) was transcriptionally upregulated upon RAS activation and rendered essential for survival and malignant transformation in ovarian epithelial cells^28^. In a murine model of Kras-G12D-driven lung adenocarcinoma, the mouse homolog of IL8 was increased in lung tissue homogenates, and treatment of these mice with an antibody targeting Cxcr2, the receptor for Il8 and Cxcl1, significantly reduced lung tumour burden^29^. High expression of IL8 was also identified in human lung adenocarcinomas with mutant KRAS, and was associated with poor clinical outcome^30^. Furthermore, increased expression and secretion of IL8 was recently shown to be essential for the induction of protease-dependent invasion and metastasis of RAS mutant melanoma cells upon BRAF inhibition^31^. The involved proteases were MMP1, PLAU, and PLAUR^31^, of which the latter two were also upregulated in MCF10A-G13D cells (Table S2). Thus, while previous investigations into the effects of mutant KRAS on cytokine signalling have primarily focused on codon 12 mutations, reflecting the substantially higher prevalence of these alleles compared to mutations affecting other codons, our gene expression data suggest that the impact of KRAS-G13D is even more pronounced. However, there may be tissue-specific differences in the effects of various KRAS mutations on cytokine signalling as well as cell-extrinsic contributions from the tumour microenvironment in mouse models and human patients that are not accounted for by our *in vitro* experimental system.

Analysis of the entire complement of genes significantly upregulated in MCF10A-G13D versus MCF10A-G12D cells using the Ingenuity Pathway Analysis tool (http://www.ingenuity.com) confirmed several of the above mentioned cellular processes and signalling cascades (Figure 4f). For example, ILK signalling includes integrins, which are a component of the gene signature associated with the basal subtype of breast cancer that was identified through GSEA (Figure 4c); IL8 signalling was also identified by STRING analysis (Figure 4d and e); and activated p53 signalling was identified through detection of phosphorylated p53 by western blotting (Figure 2c).

## Conclusions

In summary, we established isogenic cell lines that allowed characterization of eight different KRAS mutations expressed at physiological levels. Our data support the conclusion that KRAS mutations affecting different codons have distinct effects on KRAS activity, signal transduction, transcriptional programs, and transforming capacity (Table 1). The overall effects in MCF10A cells were weaker than those of KRAS variants expressed at supraphysiological levels, which allowed discrimination of biological functions related to specific mutations rather than KRAS expression level. While none of the mutations tested induced obvious morphological changes or migratory abilities, clear differences were identified in the induction of EGF-independent proliferation and anchorage-independent growth. Finally, KRAS-G13D induced EGFR expression, senescence, and a distinct pattern of transcriptional changes that may help explain the improved response of patients with G13D-positive cancers to anti-EGFR therapies.

## Methods

### Cell lines

The MCF10A cell line was obtained from the American Type Culture Collection (CRL-10317) and maintained at 37°C with 5% CO_2_. Cells were grown in DMEM/F12 supplemented with 5% horse serum, 20 ng/ml EGF, 0.5 mg/ml hydrocortisone, 100 ng/ml cholera toxin, 10 μg/ml insulin, and 1 × penicillin/streptomycin. Cells were fed twice a week and grown to 70% confluency before subculturing. Briefly, cells were washed once with PBS and incubated with trypsin for 15–20 minutes. Completely detached cells were resuspended in complete medium and centrifuged at 1,500 rpm for 3 minutes. Cell pellets were diluted 1:5.

### DNA constructs, viral transduction, and generation of cell clones

The *KRAS* cDNA in the pDONR221 vector (Invitrogen) was generated previously^32^. Sequence variants (KRAS-G12C, G12D, G12V, G13C, G13D, Q61H, A18D, and K117N) were generated using the QuikChange XL Site-Directed Mutagenesis Kit (Stratagene), and mutations were confirmed by direct sequencing. cDNAs were cloned into the pLenti6.2/V5-DEST lentiviral vector (Invitrogen). Generation of lentiviral particles and lentiviral transduction of MCF10A cells were performed as described previously^32^, and infected cells were selected with 10 μg/ml blasticidin. For the generation of clones, stably transduced MCF10A cells were diluted and seeded in 96-well plates, and wells containing only one cell were marked. Cell lines that grew out from single cells were further investigated.

### RNA isolation, cDNA synthesis, and quantitative RT-PCR

Total RNA was isolated using the RNeasy Mini Kit (Qiagen) and reverse-transcribed (2 μg in a total volume of 30 μl) using TaqMan Reverse Transcription Reagents (Applied Biosystems). *KRAS* transcript levels were measured by quantitative RT-PCR using TaqMan Gene Expression Assays Hs00364284_g1 (total KRAS) and Hs00364282_m1 (endogenous KRAS) (Applied Biosystems). Gene expression relative to endogenous *PBGD*^33^ was calculated using the standard curve method.

### Cell viability and proliferation assay

For measurement of viability and proliferation on five consecutive days, 500 cells were plated in triplicates in five 96-well plates. Each day, 20 μl CellTiter96AQ_ueous_One Solution Reagent (Promega) were added per well. After incubation for 3 hours, absorbance at 490 nm was measured using a plate reader. To determine IC50 values for the EGFR inhibitors erlotinib and gefitinib (Selleck Chemicals), 750 cells/well were plated in 96-well plates in triplicate and drugs were added the next day. Viability and proliferation were determined 48 hours after drug addition.

### Senescence assay

3 × 10^5^ cells/well were plated in triplicates in 6-well plates and cultured overnight. Cells were stained with the Senescence β-Galactosidase Staining Kit according to the manufacturer's protocol (Cell Signaling), and blue cells were counted the next day using a phase contrast microscope.

### Anchorage independence assay

For evaluation of colony formation in soft agar, 2 × 10^5^ cells were resuspended in RPMI-1640 containing 10% fetal calf serum (FCS) and 0.35% noble agar, and plated on top of a layer of RPMI-1640 containing 10% FCS and 0.5% noble agar in a 35-mm dish in triplicate. Medium was changed twice a week. After six weeks, colonies were stained with 0.005% crystal violet, counted microscopically, and photographed.

### Wound healing assay

1.2 × 10^6^ cells were grown in 6-well plates as confluent cell monolayer and starved overnight with assay medium (2% horse serum, no EGF) to minimise cell proliferation. On day 2, the cell layer was scraped with a 20 μl pipette tip in a straight line to obtain a scratch. Pictures from two different areas for each condition were taken with a phase contrast microscope every 30 minutes for 24 hours. The area of the wound at 0 and 24 hours after the initial scratch was measured with the ImageJ software program (http://imagej.nih.gov/ij/).

### Western blotting

Cells used for western blot analysis were treated under different conditions. Starved cells were maintained overnight in DMEM/F12 medium without horse serum, EGF, and insulin. Unstarved cells were cultured in complete DMEM/F12 medium containing all supplements. Whole-cell protein extracts were prepared by using lysis buffer containing 20 mM Tris-HCl, 150 mM NaCl, 5 mM EDTA, 10% glycerol, and 1% Triton X-100 supplemented with Halt Protease and Phosphatase Inhibitor Cocktail (Thermo Scientific), and 50–75 μg protein were subjected to SDS-PAGE and western blotting according to standard methods. Ponceau S staining was used to ensure equal loading. The following antibodies were used: anti-KRAS (mouse monoclonal, clone F234; 1:25 in 5% milk/TBST), anti-phospho-ERK1/2 (Y204; mouse monoclonal, clone E-4; 1:200 in 5% milk/TBST), anti-HSP90α/β (rabbit monoclonal, clone H114; 1:1000 in 5% milk/TBST) (Santa Cruz Biotechnology); anti-ERK1/2 (rabbit polyclonal, #9102; 1:1000 in 5% BSA/TBST), anti-PDK1 (rabbit polyclonal, #3062; 1:1000 in 5% BSA/TBST), anti-phospho-PDK1 (S241; rabbit polyclonal, #3061; 1:1000 in 5% BSA/TBST), anti-AKT (rabbit polyclonal, #9272; 1:1000 in 5% BSA/TBST), anti-phospho-AKT (T308; rabbit polyclonal, #9275; 1:1000 in 5% BSA/TBST), anti-phospho-AKT (S473; rabbit polyclonal, #9271; 1:1000 in 5% BSA/TBST), anti-S6K1(rabbit polyclonal, #9202; 1:1000 in 5% BSA/TBST), anti-phospho-S6K1 (T389; rabbit monoclonal, clone 108D2, #9234; 1:1000 in 5% BSA/TBST), anti-phospho-RPS6 (S240/244, rabbit polyclonal, #2215; 1:2000 in 5% BSA/TBST), Pathscan Multiplex Western Cocktail II (rabbit, #5302; 1:200 in 5% BSA/TBST), anti-RPS6 (mouse monoclonal, clone 54D2, #2317; 1:1000 in 5% milk/TBST), anti-RSK1 (rabbit monoclonal, clone D6D5, #8408; 1:1000 in 5% BSA/TBST) (Cell Signaling Technology); anti-p53 (mouse monoclonal, clone DO-7, #554294; 1:1000 in 5% milk/TBST) (BD Biosciences); anti-actin (mouse monoclonal, AM1829b; 1:2000 in 5% milk/TBST) (Abgent). Incubation with primary antibodies overnight at 4°C was followed by exposure to horseradish peroxidase-conjugated secondary antibodies (GE Healthcare).

### RAS activity assay

Cells were grown to 50–70% confluency for three days, followed by starvation in plain medium overnight. The next day, cells were stimulated with EGF (20 ng/ml) for 10 minutes or left untreated, rinsed with ice-cold PBS, and lysed in 1 × Mg^2+^ Lysis/Wash Buffer (MLB; Millipore) containing 10% glycerol and supplemented with EDTA-free Halt protease and phosphatase inhibitors (Thermo Scientific), and 500 μg protein lysate was adjusted to 500 μl with MLB. Pulldown of RAS-GTP was performed by adding 10 μl of Ras Assay Reagent (Raf-1 RBD agarose; Millipore), followed by incubation for 30 minutes at 4°C on a rotation wheel. Beads were pelleted by centrifugation at 14,000 rpm for 5 seconds and washed three times with 500 μl of 1 × MLB. Proteins were eluted by boiling in 10 μl NuPAGE sample buffer supplemented with NuPAGE sample reducing agent (Invitrogen) at 95°C for 5 minutes before subjecting to electrophoresis and immunoblotting with anti-KRAS (mouse monoclonal, clone F234; 1:25 in 5% milk/TBST) as described above.

### Gene expression profiling

Total RNA was isolated using the RNeasy Mini Kit (Qiagen), and 500 ng of quality-checked total RNA were labelled and hybridized to HumanHT-12 v4 Expression BeadChips using whole genome BeadChip Sentrix assays (Illumina). Measurements were performed in biological triplicates, and data normalisation and filtering was performed as previously reported using the BRB Array Tools software package (available at http://linus.nci.nih.gov/BRB-ArrayTools.html).

## Author Contributions

C.S. and S.F. designed the study. B.S., C.S. and S.F. wrote the manuscript, and B.S. and C.S. prepared the figures. B.S. and S.R. performed the experiments and analysed the data. L.B. and C.S. analysed the gene expression data. All authors reviewed and approved the manuscript.

## Additional information

The complete microarray data are available at the Gene Expression Omnibus (http://www.ncbi.nlm.nih.gov/projects/geo; accession number GSE60695).

## Supplementary Material

Supplementary Information Supplemental Figures

Supplementary Information Supplemental Table S1

Supplementary Information Supplemental Table S2

## Figures and Tables

**Table 1:** Effects of diverse KRAS mutations expressed at physiological levels in MCF10A cells

	WT	G12C	G12D	G12V	G13C	G13D	A18D	Q61H	K117N
**KRAS-GTP**	−	+	−	+	+	−	−	++	++
**Pathway activation**#									
• **EGFR**	−	−	−	−	++	+++	−	−	−
• **p-p53**	−	−	−	−	−	+++	−	−	−
**Morphology**	−	−	−	−	−	−	−	−	−
**EGF-independent proliferation**	−	−	+++	++	−	+++	−	−	++
**Anchorage-independent growth**	−	+	−	+	−	−	−	++	+++
**Migration**	−	−	−	−	−	−	−	−	−
**Transcriptional changes in G13D versus G12D**	−	ND	Luminal BC	ND	ND	Basal BC	ND	ND	ND
						Cytokine signalling up			
						p53 signalling up			

Minus sign indicates no observed effect.

Plus sign indicates observed effect.

ND, not done.

BC, breast cancer subtype.

^#^As determined by western blotting; only signalling proteins with observed effects under starved conditions are listed.
