# Supplementary material for: Comparative analysis of KRAS codon 12, 13, 18, 61, and 117 mutations using human MCF10A isogenic cell lines
Source: Sci Rep. 2015 Feb 23;5:8535. doi: 10.1038/srep08535 (PMC4336936; doi:10.1038/srep08535)
Supplement: Supplementary Information — Supplemental Figures [file srep08535-s1.pdf]

Supporting Online Information for

Comparative analysis of KRAS codon 12, 13, 18, 61, and 117 mutations using human MCF10A isogenic cell lines

Britta Stolze, Stefanie Reinhart, Lars Bulllinger, Stefan Fröhling, Claudia Scholl

Supplemental Figure 1

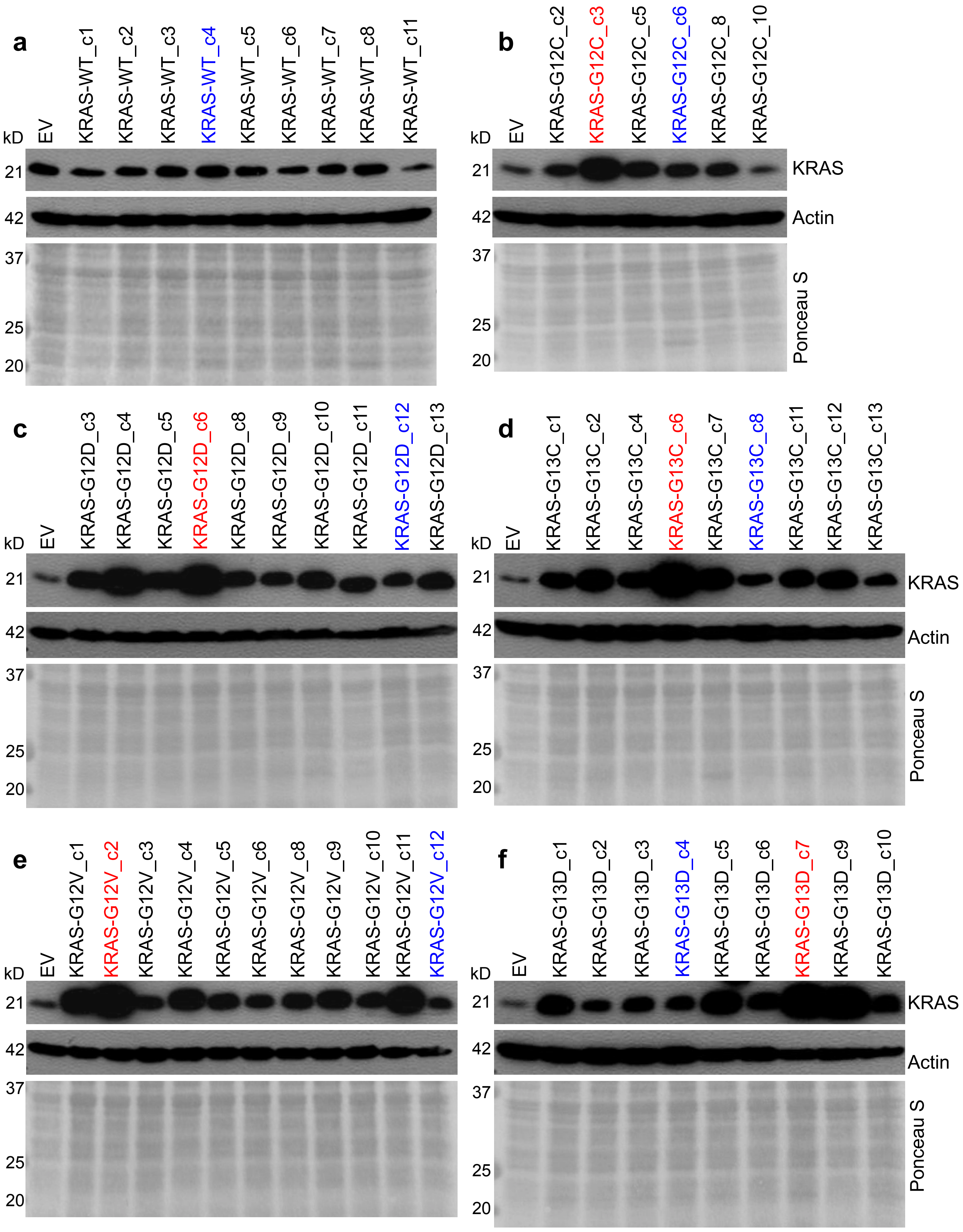

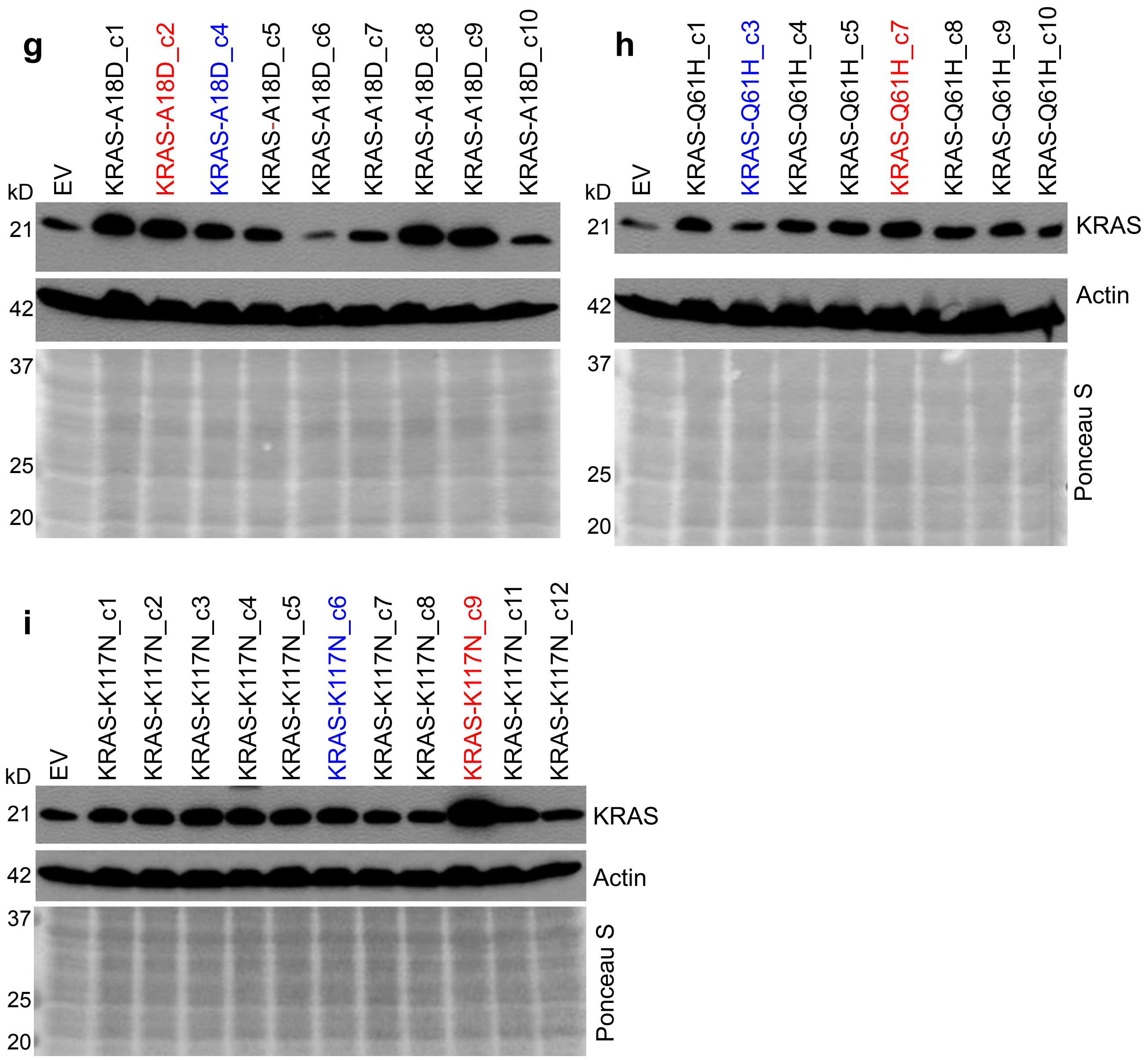

Figure S1. KRAS protein expression in EV-transduced MCF10A cells and MCF10A clones expressing WT KRAS or various KRAS mutations. (a-i) Western blot analysis of KRAS protein expression in established MCF10A clones expressing WT KRAS (a), KRAS-G12C (b), KRAS-G12D (c), KRAS-G13C (d), KRAS-G12V (e), KRAS-G13D (f), KRAS-A18D (g), KRAS-Q61H (h), and KRAS-K117N (i) compared to MCF10A cells transduced with EV. The clones highlighted in blue and red were selected as low- and high-expressing clones, respectively. Actin expression and Ponceau S staining indicate equal loading.

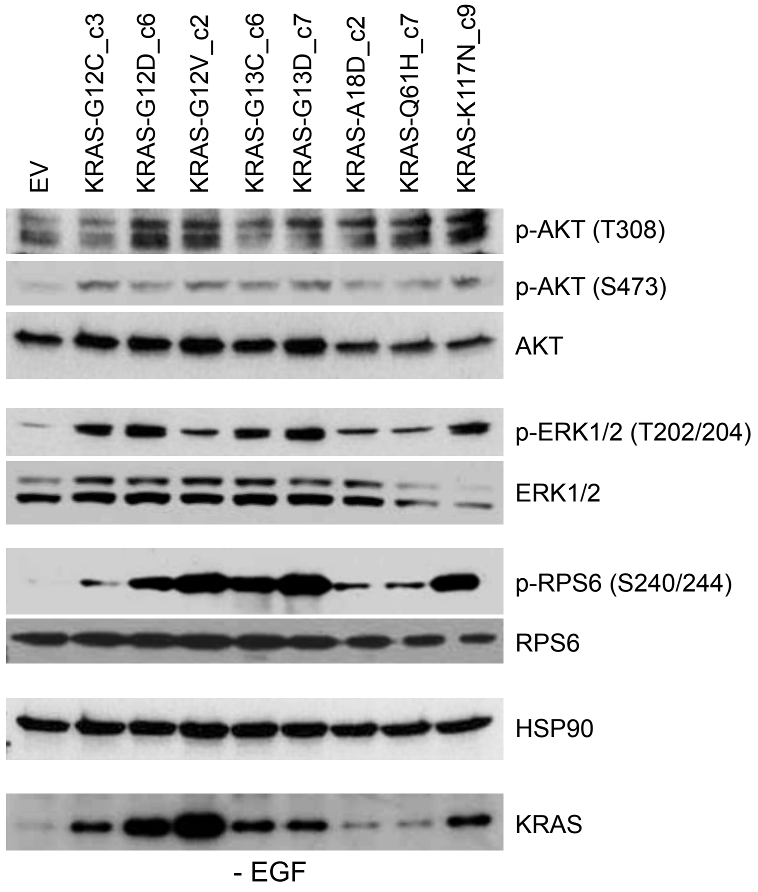

Figure S2. Effect of KRAS mutations expressed at high levels on downstream signaling proteins. Western blot analysis of MCF10A clones expressing high levels of different KRAS mutations and cultured without EGF overnight. HSP90 expression indicates equal loading. One of at least two independent experiments is shown.

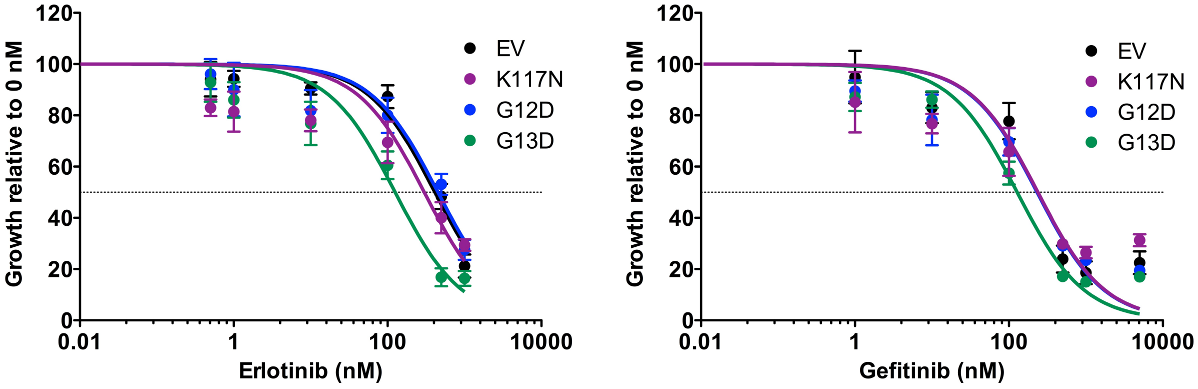

	EV	K117N	G12D	G13D
IC50	412.5	300.2	451.7	125.4

	EV	K117N	G12D	G13D
IC50	235.3	237.5	231.8	127.8

Figure S3. Effect of KRAS mutations on EGFR inhibitor sensitivity. Dose-response curves of MCF10A clones expressing low levels of different KRAS mutations after treatment with erlotinib and gefitinib for 48 hours. IC50 values were calculated from three independent experiments performed in triplicate (mean ± SEM). Dose response curves and IC50 values (nM) are shown.

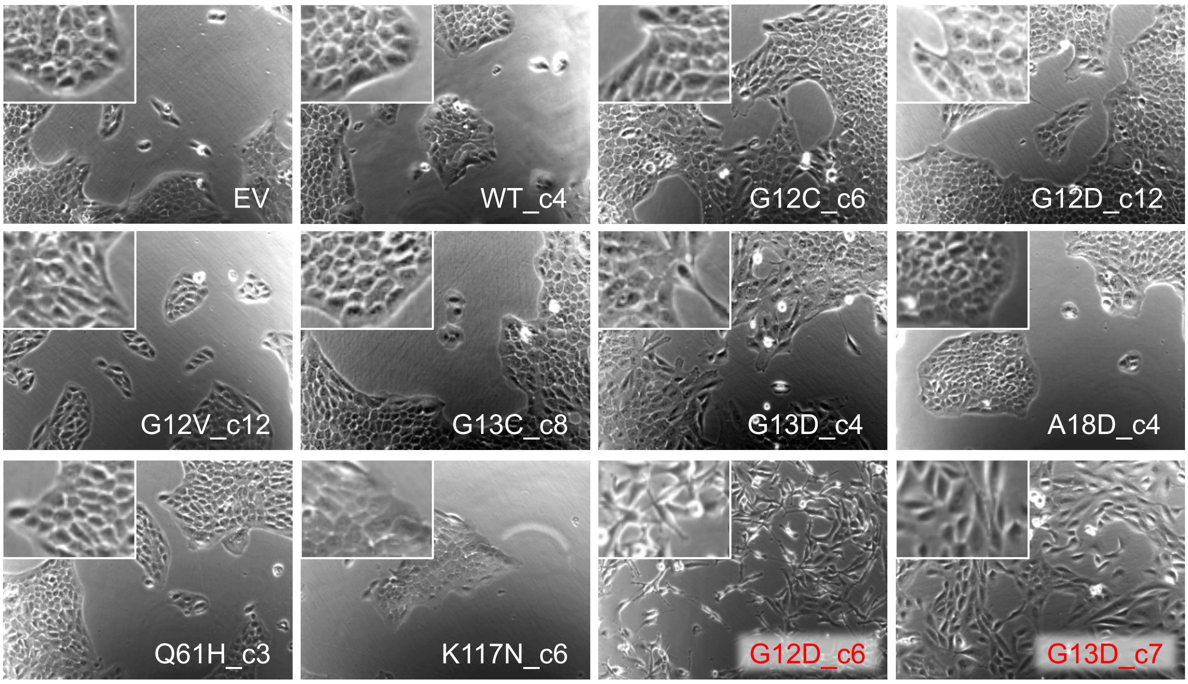

Figure S4. Effects of KRAS mutations on cell morphology under starved conditions. MCF10A clones expressing the indicated KRAS mutations at low (white letters) and high (red letters) levels were grown to 50% confluency and starved overnight. Representative phase contrast micrographs are shown (original magnification, 10x).

a

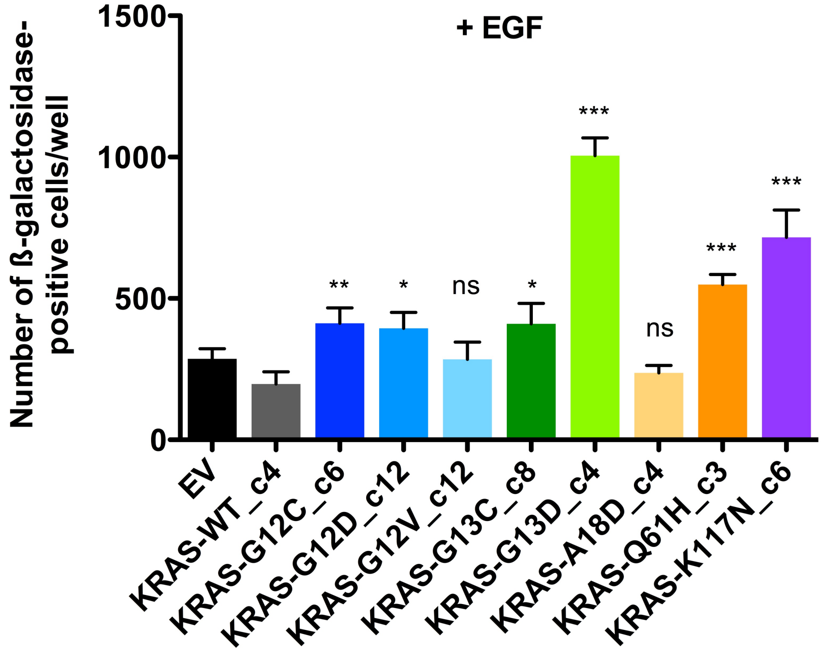

b

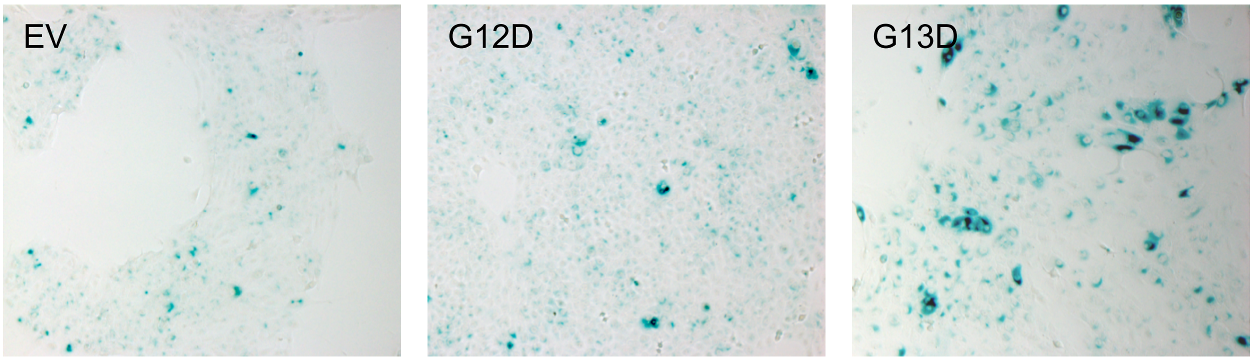

Figure S5. Effects of KRAS mutations on senescence. (a) β-galactosidase positivity of MCF10A clones expressing different KRAS mutations or KRAS WT at low levels and EV-transduced MCF10A cells. Results of two independent experiments performed in triplicate are shown (mean ± SEM). Numbers of ß-galactosidase-positive cells were compared between clones expressing mutant KRAS and clones transduced with WT KRAS or EV. P values were calculated using an unpaired t-test. (b) Representative phase contrast micrographs of the indicated MCF10A clones are shown (original magnification, 10x).
*, $P < 0.05$; **, $P < 0.01$; ***, $P < 0.0001$; ns, not significant.

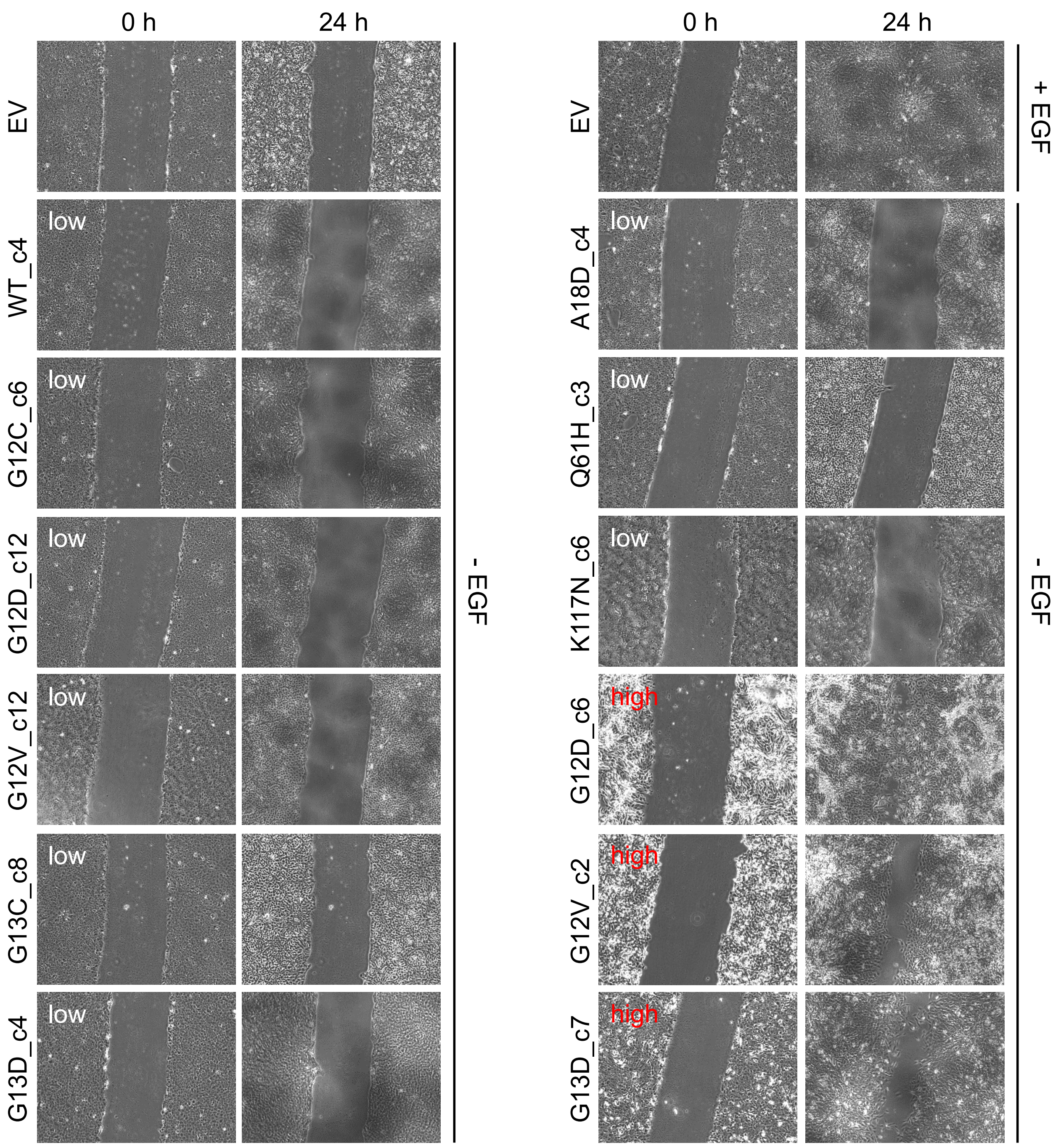

Figure S6. Effects of KRAS mutations on cell migration. Representative phase contrast micrographs (original magnification, 4x) of MCF10A clones expressing the indicated KRAS mutations at low (white letters) and high (red letters) levels in a wound healing assay. A 100% confluent monolayer was starved overnight, and photographs were taken immediately after scratching and after 24 hours. EGF addition (+ EGF) or withdrawal (- EGF) during the 24-hour incubation is indicated.
